# Microbial Risk Assessment Framework for Exposure to Amended Sludge Projects

**DOI:** 10.1289/ehp.10994

**Published:** 2008-03-13

**Authors:** Joseph N.S. Eisenberg, Kelly Moore, Jeffery A. Soller, Don Eisenberg, John M. Colford

**Affiliations:** 1 Department of Epidemiology, University of Michigan, Ann Arbor, Michigan, USA; 2 School of Public Health, University of California Berkeley, Berkeley, California, USA; 3 Soller Environmental, Berkeley, California, USA; 4 EOA Inc, Oakland, California, USA

**Keywords:** environment, microbial, risk assessment, sludge

## Abstract

**Background:**

Although the U.S. Environmental Protection Agency has a long history of using risk-based approaches for regulatory purposes, pollutant limits for pathogens in biosolids are not currently based on quantitative risk assessments.

**Objectives:**

We developed and demonstrated a risk-based methodology for assessing the risk to human health from exposure to pathogens via biosolids.

**Materials:**

Four models were developed, incorporating direct ingestion, groundwater, and aerosol exposure pathways. Three sources of environmental data were used to estimate risk: pathogen monitoring of sludge, efficacy of sludge treatment, and pathogen monitoring of biosolids.

**Results:**

Risk estimates were obtainable even for Class A biosolids, where posttreatment monitoring data are below detectable levels, demonstrating that risk assessments for biosolids exposure are practical. Model analyses suggest that: *a*) a two-digester design decreases the probability of risks > 10^−4^ compared with one-digester designs, *b*) risks associated with exposures to groundwater and aerosol pathways were, in general, lower than exposures to the direct ingestion pathway, and *c*) secondary transmission can be an important factor in risk estimation.

**Conclusions:**

The risk-based approach presented here provides a tool to *a*) help biosolids producers interpret the results of biosolids monitoring data in terms of its health implications, *b*) help treatment plant engineers evaluate the risk-based benefits of operational changes to existing or projected treatment processes, and *c*) help environmental managers evaluate potential capital improvements and/or land application site placement issues. Regulation of pathogens can now be based on human health risk in a manner parallel to other water-related risks.

Treated sewage sludge, referred to as biosolids, has been used as a soil amendment for agricultural purposes since the onset of municipal wastewater treatment, and its use has intensified over the past 20 years. In 1993, the U.S. Environmental Protection Agency (U.S. EPA 1993) published the Part 503 rule, which is designed to protect the public’s health and the environment from adverse effects of pollutants that may be present in biosolids (U.S. EPA 1995). In the Part 503 rule, pollutant limits for chemical constituents in biosolids were based on the results of risk assessments that were conducted to identify what, if any, risks were associated with the use or disposal of biosolids. Pollutant limits for pathogens, however, were not based on risk assessments; rather, they were based on performance, technology-based standards, or management and record-keeping practices intended to protect human health and the environment. At the time, the U.S. EPA thought that methodologies had not been developed sufficiently to make risk-based calculations, especially given the lack of exposure data and limitations in analytical methods (U.S. EPA 1989, 1992, 1995). Here we present an approach to conducting risk assessments associated with biosolids exposures, given the improved but still minimal availability of exposure data. The model as demonstrated herein provides the necessary framework for microbial risk assessment, both providing risk estimates and identifying data gaps that, when filled, will provide more robust estimates.

The U.S. EPA has a long history of using risk assessment to inform its regulatory decision making, specifically to address their mandate to protect the public from environmental exposures to waterborne enteric pathogens. The use of risk assessment to address concerns about water-related risks has originated partly from well-documented outbreaks associated with drinking water and recreational water exposures. Additional concern has been the result of studies of risks in non-outbreak conditions. For example, numerous prospective studies have shown a link between fresh and marine water exposure through swimming and gastrointestinal illness. Although no documented scientific evidence indicates that the current Part 503 has failed to protect the public’s health, polarization among advocates and critics of the use of biosolids has increased in recent years, and this has raised questions about the effectiveness of the current pathogen standards for protecting the public. In response to this polarization, the U.S. EPA requested the formation of a National Research Council (NRC) committee to review the adequacy of the regulations, including the technical basis of the pathogen requirements for biosolids and the feasibility of using microbial risk assessment. The conclusions reached by the committee were that

there is no documented scientific evidence that the Part 503 rule has failed to protect public health. However, additional scientific work is needed to reduce persistent uncertainty about the potential for adverse human health effects from exposure to biosolids. (NRC 2002)

In this article we extend previously published risk assessment models, using scenario-specific information from Brooks et al. (2005) and Eisenberg et al. (2004), to the analysis of pathogen data from biosolids. The current model extends these studies in a number of ways. First, it couples together three data sources: pathogen data from raw sludge, treatment process efficacy data, and post treatment pathogen monitoring data. The use of all three data sources provides a more robust approach to risk estimation, in that it allows for the estimation of risk even when posttreatment pathogen data are all below the detectable level. The incorporation of raw sludge data also provides a way to examine the effects of variation in influent concentration on the risk estimate. Explicit modeling of the treatment process allows for the evaluation of the health impact due to environmental changes in the context of an existing treatment process or due to design improvements to the treatment process. Furthermore, even if the posttreatment monitoring data are below detectable levels, modeling ensures that risk estimates are consistent with these nondetectable pathogen data. Second, the current model examines three exposure pathways (direct, groundwater, and aerosolized) as well as two population groups (residential and occupational). Models for transport through groundwater and air are provided. And finally, our model provides risk estimates incorporating both a more streamlined individual-level risk model, akin to chemical risk models, and a dynamic population-level model that accounts for secondary transmission and immunity.

## Methods

For this analysis, the risk assessment model can be thought of as a two-step process. First, an exposure model estimates the exposure dose for humans; then a health effects model is used to estimate risk.

### Exposure model

**Figure 1:**
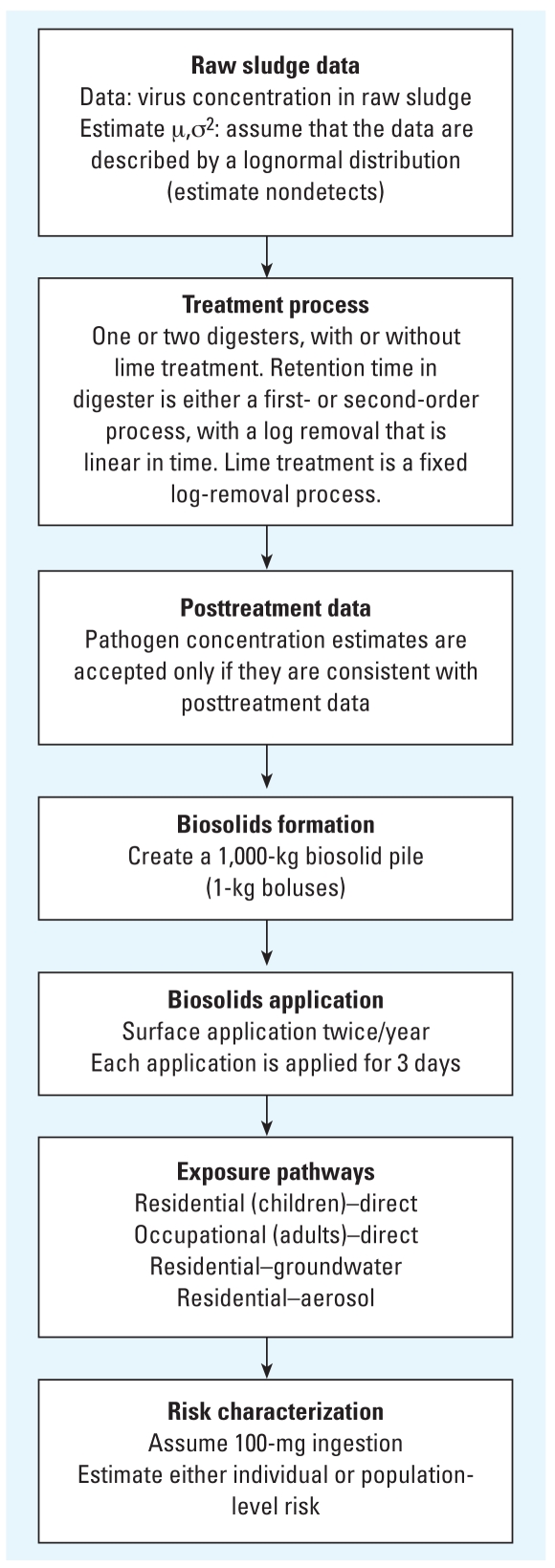
General model flow diagram that includes exposure assessment and risk characterization.

The exposure model can be broken down into five components (Figure 1).

#### Modeling enteric virus concentrations in raw sludge

Data are fit to a lognormal distribution, using a regression on order statistics (ROS) technique to impute nondetectable values (Shumway et al. 2002) [See Supplemental Material: Appendix 1 for details (online at http://www.ehponline.org/members/2008/10994/suppl.pdf)].

#### Modeling the treatment process

The model used in this analysis includes a combination of either one or two mesophilic, anaerobic digesters with or without lime treatment. By assuming that the sludge in the digester is well mixed, the retention time in the treatment digester can be modeled as a first-order process: It follows an exponential distribution. The mean log removal of the whole biosolids train is therefore constant, but the specific log removal for a given bolus will vary depending on its retention time. The log-removal is assumed to be linearly related to retention time. When using two digesters in series, the overall time becomes a gamma distribution; that is, it is the sum of two exponentials, β_ret1_ + β_ret2_. Lime treatment is assumed to be a fixed log-removal process.

#### Modeling pathogen concentrations in biosolids (posttreatment)

Posttreatment monitoring data are used to constrain exposure estimates; predicted pathogen concentrations from the treatment process model should be consistent with these monitoring data. Monitoring data from Class A biosolids, which by definition must all be below the detectable limit of 1 plaque-forming unit (PFU) per 4 g, necessarily contain posttreatment concentrations below the detectable limit. For Class B biosolids, the condition is not as strong, and there may be detectable concentrations even after treatment. This is reflected by a less stringent boundary condition. In all cases, the boundary condition used is based on the measured posttreatment concentrations. To demonstrate the model, we assume a Class A process so that the measured output is always assumed to be below detectable levels. Monitoring, however, is limited in both space and time. The boundary condition applied here, therefore, requires that concentration levels are below detectable levels 99% of the time. The value of 99% simply reflects the fact that if 1% of the samples are above the detection limit, detecting viruses in a standard monthly, single grab sample will be rare. Analytical limitations, including the ability to detect only a subset of all pathogenic viruses, may argue for a less stringent boundary condition, but for this demonstration of a risk assessment and the limited data to inform this condition, we use 99% in these analyses.

#### Modeling the biosolids pile

For each simulation, a biosolids pile is created 1 kg at a time. The viral concentration in each 1-kg bolus will depend on the retention time, which is a stochastic process, and will therefore vary from bolus to bolus. In this manner, the variability of the digester process will determine the spatial distribution of the viral concentrations in the biosolids pile.

#### Modeling exposure pathways

In the scenario used for this risk assessment, the biosolids are assumed to be applied to the surface of agricultural land twice per year, where each application lasts for a period of 3 days. We can define a few possible routes of exposure. First, two groups may be exposed via direct ingestion. Children in residences near the application site are exposed during the application period, and workers involved in the treatment process or application are exposed 5 days a week. Second, nearby residences are exposed when their inhabitants drink ground-water contaminated with pathogens from the biosolids. Residences nearby may also be exposed through aerosol transport.

### Exposure data

Monitoring data for raw sludge was collected from a variety of utilities. In general, these data sets consist of a full year of monthly enteric virus data, with a 1 PFU/4 g dry weight detection limit. For most utility monitoring sites, the analysis of samples in the treated sludge resulted in enteric virus levels below the detection limit, primarily because most utilities that have enteric virus data are producing Class A biosolids. For the risk assessment presented here, we assumed that all treated sludge data are nondetects, thus providing a model for Class A biosolids. Table 1 provides the monitoring data used in this analysis, 1 year of monthly samples from an anonymous utility.

Table 2 provides a brief summary of the treatment data available in the literature to estimate the log removal for anaerobic digestion. The first five estimates of the total log10 reduction for mesophilic digestion range from 0.5 to 2. The table provides one reference for the log10 reduction per day at three plants, which range from 0.003 to 0.03. The only thermophilic digestion data source provides an estimated log10 reduction of 3. The final reference provides an equation of inactivation per day and inactivation of viruses based on temperature. Based on these data sources, an estimate of 2-log is used in the model as the mean attenuation of viruses during 15 days of anaerobic digestion in each of the risk assessment models.

The data collected from the literature for soil ingestion ranged from 26 to 480 mg/day [see Eisenberg et al. (2006) for a more detailed summary of these studies]. The high-end estimate of 480 mg/day corresponds to an adult performing 8 hr yard work twice per week. Based on these data, the direct ingestion rate used in each of the risk models was 100 mg/day.

### Definitions of risk

Three types of risks were estimated from the models: individual-level single-event risk, individual-level annual risk, and population-level attributable risk. All three risk measures are based on the beta-Poisson dose–response function that estimates the probability of infection given a specified dose:


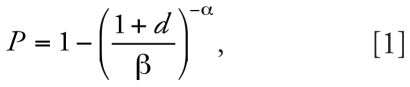


where *P* represents the probability that an exposed individual will become infected, *d* represents the dose, and β and α are parameters for a specific virus. The dose is equal to the product of the *a*) concentration at the exposure site (pathogens/volume), *b*) rate of ingestion (volume/time), and *c*) time of exposure (time). Here, volume is represented as grams dry weight. The virus used in all risk models is rotavirus with β = 0.42 PFU and α = 0.26 (unitless) (Regli et al. 1991).

The individual-level single event risk is defined as the risk associated with an individual’s one-time exposure to biosolids (equal to *P* in Equation 1). The exposure or dose is estimated using one of the models described in the following sections and an ingestion rate for the given exposure pathway. For example, an individual-level risk of 3 × 10^−3^ means that for every 1,000 times an individual is exposed to this dose, he or she will likely become infected or sick three times.

An annual individual-level risk is defined as the risk associated with an individual’s exposure to biosolids over a year-long period. It is calculated from the individual-level risk as


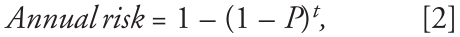


where *P* is the individual-level risk and *t* is the time exposed in days. Based on the previous example and assuming an occupational exposure with a 5-day work week, and thus 260 days of exposure per year, the annual individual-level risk is 0.54. This means that a worker exposed to these conditions every day of his work year can expect to become infected or sick approximately 0.54 times in any given year.

Population-level annual attributable risk is based on a standard population-level measure used in epidemiology, the cumulative incidence (CI), which is defined as the number of new cases of disease during a 1-year period divided by the total population at risk. To obtain a measure of population risk, simulations are run twice—once with biosolids exposure present and once with biosolids exposure absent—to obtain CI_b_ and CI_0_. The attributable risk (AR) due to biosolids is then calculated as the difference between CI_b_ and CI_0_. For example, let’s say that when biosolids are present, the model produces CI_b_ = 1.56 cases per person per year, and when biosolids are absent, the model produces CI_0_ = 1.44 cases per person per year. The attributable risk due to biosolids would be AR = 0.12 cases per person per year. In this example, the number of cases due to biosolids is a small percentage of the total cases.

We used the dose–response function to estimate the risk of a given dose of viruses. However, the risk in the population model is also based on risk from person-to-person transmission in addition to the risk associated with ingestion of viruses.

### Risk assessment models

We developed four models to examine the issues associated with risks. See the Supplemental Material [Appendix 2 (online at http://www.ehponline.org/members/2008/10994/suppl.pdf)] for details on the simulation approach of these models and Table A-2 for a summary of the major assumptions of our model.

**Figure 2:**
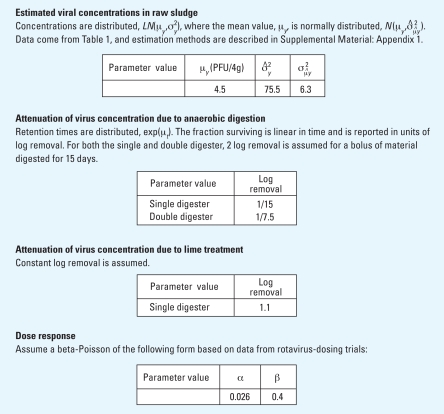
Summary of parameter values for risk models 1 and 2.

- The first model estimates individual-level risk for direct ingestion. The exposure model uses one anaerobic digester with a mean retention time of 15 days, and is otherwise structured as described in the previous exposure model subsection.
- The second model is identical to the first except for the addition of a second digester placed in series, where each digester now has a mean retention time of 7.5 days. The total mean retention time over the two digesters is 15 days. Figure 2 provides a summary of parameter values for risk models 1 and 2.
- The third model is identical to the first model with the addition of either a ground-water [Supplemental Material: Appendix 3 (online at http://www.ehponline.org/members/2008/10994/suppl.pdf)] or aerosol [Supplemental Material: Appendix 4 (online at http://www.ehponline.org/members/2008/10994/suppl.pdf)] pathway. The groundwater model allows for the calculation of risks based on exposure to contaminated groundwater for a number of different soil depths and types. Specifically, we modeled three soil types: nonporous, saturated, and unsaturated. Using these three soil types, we ran risk assessments for 10 scenarios: nonporous soil, nonporous soil followed by 5, 15, or 30 m of saturated soil, and 0.25 or 0.5 m of unsaturated soil followed by 5, 15, or 30 m of saturated soil. The aerosol model likewise addresses the issue of exposure to biosolids pathogens that have been transported by wind. Specifically, it includes wind speeds of 2, 5, and 10 m/sec at downwind distances from the biosolids application site of 30, 100, and 250 m.
- The fourth model extends the first model to estimate population-level risks. Specifically, this model examines the role of both direct exposure to environmental contamination and indirect exposure due to secondary transmission. We used a previously developed dynamic model to estimate population-level risks (Eisenberg et al. 2004). See Supplemental Material: Appendix 5 (online at http://www.ehponline.org/members/2008/10994/suppl.pdf) for the simulation details of model 4.

Risk estimates for models 1 and 2 were all single-event risks. Single-event risks provide information on the probability of infection or disease of an individual that is exposed to biosolids. For both the groundwater and aerosol models, we estimated annual risks for residential exposure along with single-event risks, based on a 3-day exposure per application for biosolids applied two times per year. The annual risk estimates take into account the amount of biosolids exposure during a given year as well as the target population that is exposed. Therefore, the annual risks can provide information on the health cost to the population. Although the annual risks were only calculated for model 3, they can be used as the risk measure for models 1 and 2.

The annual risk estimates assume that the disease outcome of one individual is independent of the disease outcome of other individuals (Eisenberg et al. 2006). Therefore, it is still an individual-level analysis that is scaled up to provide population estimates. When accounting for secondary transmission, a full-fledged population analysis is required; this is the focus of model 4. Using model 4 requires a population measure of risk; therefore, single-event risk estimates are not appropriate. Rather, annual risks are estimated using an attributable risk metric: the number of cases of infection or disease that are attributable to biosolids exposure. For these simulations, therefore, the residential scenario assumes a 3-day exposure per application for biosolids applied two times per year. Occupational exposures assume workers are exposed 5 days per week, 52 weeks per year. Comparing the residential and occupational exposures, residential exposures dominated the overall risk estimate because the target population was so much larger, even though the yearly exposure in the occupational setting was larger. Given the scope of this work, the comparison of these two exposure scenarios was not meant to imply the importance of one exposure over the other; rather, it was meant to demonstrate that the role of a risk assessment is to provide estimates for a variety of scenarios so that those involved in risk management can use this information for decision making.

## Results

**Figure 3:**
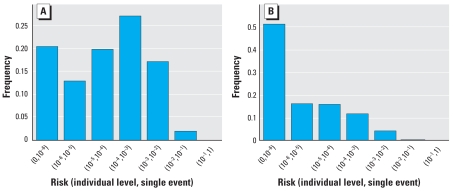
Distribution of risk with additional lime treatment. (*A*) Model 1 with 1.1 log removal from lime treatment; mean = 1 × 10^−3^, σ = 3 × 10^−3^. (*B*) Model 2 with 0.5 log removal from lime treatment; mean = 2 × 10^−4^, σ = 1 × 10^−3^.

Table 2 summarizes the parameters used in models 1 and 2. The resulting risk estimates from model 1 (single digester) and model 2 (double digester) are shown in Figure 3, and a comparison of these two models is shown in Table 3. Compared with one digester, the mean risk is lower for the two digesters (2 × 10^−4^ vs. 1 × 10^−3^). Retention times are also shorter compared with the one-digester model, and the variability, as measured by the standard deviation, in the risk estimates for the two-digester model is lower than that of the one-digester model (1 × 10^−3^ vs. 3 × 10^−3^). The probability of a risk > 10^−4^ is almost three times as high for the one-digester model (0.46) as compared with the two-digester model (0.16).

In model 3, the groundwater exposure simulations were run for every combination of the 10 groundwater scenarios with the one digester treatment model. The additional 1.1 log removal corresponding to lime treatment was also included.

The worst-case groundwater scenario is the nonporous media (fractured bedrock or karst) scenario. This scenario is essentially a direct conduit to the well, and therefore provides little attenuation relative to other media. Under this exposure scenario, the mean risk was 2 × 10^−2^. This risk is higher than that resulting from direct ingestion with lime treatment, which had a mean risk of 1 × 10^−3^. A reason for this difference is that the direct ingestion is based on a 100-mg sample of the 1,000-kg biosolids pile. Thus, only a small number of viruses are actually ingested. However, with the nonporous groundwater scenario, even though there is additional attenuation, leaching, and dilution, well water contains an aggregate of contamination from the biosolids applied to the land. Thus the number of viruses ingested is actually higher than in the direct ingestion scenario. When the nonporous medium was followed by saturated soil, the attenuation increased. The mean risk for this scenario was reduced to 2 × 10^−3^, 2 × 10^−5^, and 5 × 10^−8^, for 5, 15, and 30 m of saturated soil, respectively.

For the scenarios that include unsaturated soil followed by saturated soil, as the barrier depths increase, the mean risk decreased to as low as 6 × 10^−9^ for the scenario of 0.5 m of unsaturated soil followed by 30 m of saturated soil.

**Figure 4:**
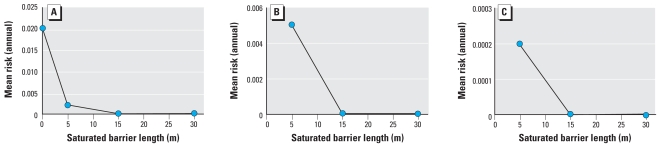
Mean risk against barrier depth (groundwater scenario). (*A*) Nonporous media followed by saturated soil. (*B*) Unsaturated soil (0.25 m) followed by saturated soil. (*C*) Unsaturated soil (0.5 m) followed by saturated soil.

A summary of the risks associated with these various models as a function of the saturated zone barrier depths is provided in Figure 4. Figure 4A provides the mean risk against the saturated barrier depth, which is preceded by nonporous media. The pattern is nonlinear: The decrease in mean risk is larger for small barrier depths compared with large barrier depths. Figures 4B and 4C provide the mean risk as a function of saturated barrier depth when preceded by unsaturated soil of depths 0.25 and 0.5 m, respectively. A similar nonlinear pattern in mean risk is observed for both of these scenarios. Thus, for the ground-water exposure, the mean risks range from 2 × 10^−2^ to 6 × 10^−9^ for the worst- and best-case scenarios, respectively. This corresponds to a single-event risk. The corresponding estimated annual risks based on 3 days exposure for biosolids applied two times in the year range from 1 × 10^−1^ to 4 × 10^−8^.

**Figure 5:**
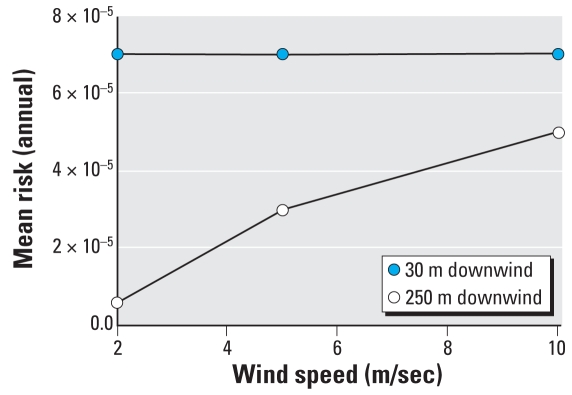
Mean risk against wind speed (aerosol scenario). Downwind distance = 30 m. Downwind distance = 250 m.

We ran the aerosol exposure simulations for a variety of aerosol scenarios for different wind speeds and downwind distances with the first-order digester treatment model and additional lime treatment. The results for the 30-m and 250-m downwind distances are summarized in Figure 5. In general, as the downwind distance from the source increases, the attenuation increases and thus the mean risk decreases. Similarly, as the wind speed increases, the attenuation decreases and therefore the mean risk increases. For a downwind distance from the source of 30 m, the wind speed does not have any effect on the mean risk (Figure 5A). After rounding the risk estimates, there is no observable difference for the three wind speeds. This occurs because the exposure site is quite close to the source and thus the effect of wind speed is small compared with larger downwind distances.

Increasing the distance from 30 m to 250 m has a more noticeable impact on the mean risk as it ranges from 6 × 10^−6^ at a wind speed of 2 m/sec to as high as 5 × 10^−5^ at 10 m/sec. Figure 5B shows that the increase in mean risk with wind speed is nearly linear. Thus, at longer downwind distances, the wind speed has a larger effect on attenuation and thus risk. Based on an individual mean risk of 7 × 10^−5^, assuming that biosolids are applied twice per year with a 3-day 8-hr exposure each time, the corresponding mean annual risk is 7 × 10^−4^. Similarly, the lowest risk scenario at 250 m downwind at 2 m/sec wind speed corresponds to a mean annual risk of 3 × 10^−5^.

The parameter estimates for background levels of pathogens and the secondary transmission parameter were estimated using the method outlined in Supplemental Material: Appendix 5 (online at http://www.ehponline.org/members/2008/10994/suppl.pdf) to achieve a background incidence level of 40 cases per 100,000 people per year.

**Figure 6:**
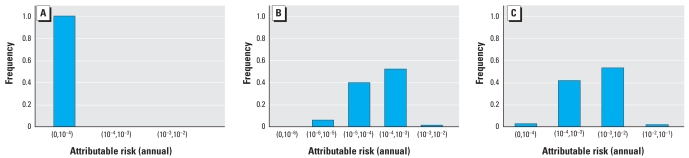
Population model attributable risk from biosolids. (*A*) Occupational only exposure, mean = 8 × 10^−5^, σ =2 × 10^−5^. (*B*) Residential only exposure, mean = 3 × 10^−3^, σ = 4 × 10^−3^. (*C*) Occupational and residential exposure, mean = 3 × 10^−3^, σ = 4 × 10^−3^.

**Figure 7:**
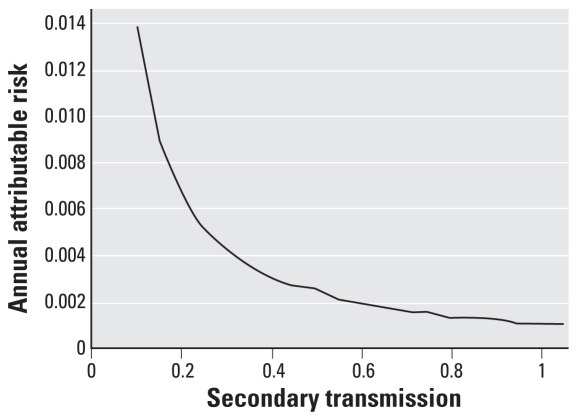
Annual attributable risk from biosolids as a function of secondary transmission.

Each of the three scenarios was simulated with 10,000 iterations each. For the first scenario with occupational exposure only, the simulation was run with and without biosolids present to estimate the attributable risk for biosolids. Figure 6 provides the attributable risk distribution for this scenario. The mean annual attributable risk was 8 × 10^−5^. For comparison, using model 1 with lime treatment, the individual-level single-event risk was 1 × 10^−3^, which corresponds to an annual risk of 2 × 10^−4^, assuming that the occupational risk applies to 0.1% of the population with 260 days of exposure per year. The main difference between the annual risk using model 1 and the annual attributable risk using model 4 is that model 4 includes secondary transmission. This comparison, therefore, suggests that secondary transmission attenuated the risk from 2 × 10^−4^ to 8 × 10^−5^, a reduction of 60%. In general, when simulations are run for increasing values of secondary transmission, the mean annual attributable risk due to biosolids decreases (Figure 7). That is, secondary transmission becomes protective, because increases in secondary transmission result in increases in infection rates due to the other environmental pathways: More people are sick in general, so less overall illness can be attributed to biosolids exposure.

We ran the second scenario again with residential exposure with and without biosolids present to estimate the attributable risk for biosolids. The residential scenario applies to children only. In this scenario, the proportion of children was set to 50% of the population. Figure 6B provides the attributable risk distribution, which has a mean annual attributable risk of 3 × 10^−3^.

The third population-level scenario combines both the residential exposure from scenario 1 and the occupational exposure from scenario 2. Figure 6C provides the attributable risk distribution, which has a mean annual attributable risk of 3 × 10^−3^. Most of the attributable risk for biosolids, therefore, arises from the residential population (3 × 10^−3^ vs. 8 × 10^−5^), because the occupational population is a very small percentage of the whole population [see Supplemental Material: Appendix 5 (online at http://www.ehponline.org/members/2008/10994/suppl.pdf)]. With secondary transmission set to zero, the attributable risk is 2 × 10^−3^ (vs. 3 × 10^−3^, a reduction of 66%).

## Discussion

Microbial risk assessment is a structured approach to estimating risks associated with environmental exposure to pathogens by using available environmental data and practical assumptions. In this study we used three sources of environmental data to estimate risk: pathogen monitoring of raw sludge, efficacy of treatment processes, and pathogen monitoring of posttreatment biosolids. We demonstrated that this risk assessment approach is able to estimate risks even for Class A biosolids where posttreatment monitoring data are all below the detectable level. Even though analytical data are limited, the methodology described herein provides a reliable model for performing risk assessment, demonstrating that comprehensive risk assessments for biosolids exposure are practical. As more data become available, the model outputs will likewise become more robust.

The first two models focus on how variability and uncertainty affect risk estimates. Presenting risk estimates as distributions rather than point estimates provides a way to evaluate how various sources of variability and uncertainty impact risk. There are many sources of variability. For example, the lognormal distribution describing the concentration of pathogens in the raw sludge represents the variability in concentration levels due to a variety of environmental and wastewater treatment process factors. The uncertainty stemming from the sample size of the monitoring data is represented by the truncated normal distribution of the mean pathogen concentration.

Models 1 and 2 account for this variability by explicitly modeling the treatment process. We assume that the digesters are well mixed, and therefore are modeled as first-order processes: At any given time, any bolus within the digester is equally likely to leave as biosolids, independent of its residence time. Compared with model 1, by adding a second digester in model 2 we observe both a lowering of the mean risk by 80% (1 × 10^−3^ vs. 2 × 10^−4^ ) and a decrease in the probability of there being a risk > 10^−4^ (0.46 vs. 0.16). This comparison demonstrates how the design of treatment processes can lower the risk by decreasing the variability in the treatment process.

Model 3 focused on risk estimates associated with two exposure pathways: groundwater and aerosol. The risks associated with the groundwater pathways were in general lower than the direct ingestion scenarios, except for the nonporous model that predicted a mean risk of 2 × 10^−2^ compared with 2 × 10^−3^ for the analogous direct ingestion estimate. Adding saturated barriers lowered the risk from 2 × 10^−3^ to 2 × 10^−7^ depending on the barrier depth. Unsaturated barriers added even more attenuation, resulting in a risk as low as 6 ×10^−9^ depending on the barrier depth.

The risk from aerosol exposure is a function of the downwind distance from the exposure site to the source and of the wind speed, and was also lower than that from the direct ingestion pathway. Risks ranged from 7 × 10^−5^, when the source was 30 m from the exposure site, to 5 × 10^−5^, when the source was 250 m away with a wind speed of 10 m/sec, to 6 × 10^−6^, when the source was 250 m away with a wind speed of 2 m/sec. The addition of secondary transmission in model 4 resulted in an attenuation of risk. This attenuation is a known feature for pathogens with multiple transmission pathways. The relative importance of a population perspective that incorporates secondary transmission depends on several factors including pathogen infectivity and the magnitude of pathogen concentrations at the exposure site (Soller and Eisenberg 2008).

A transparent risk assessment process is essential. That being said, although transparency is difficult to provide, we have attempted to articulate all of our assumptions [see Table A-2 in the Supplemental Material (online at http://www.ehponline.org/members/2008/10994/suppl.pdf)]. The balance between providing increased realism and providing a tractable model is difficult in any risk assessment. We hope that the framework we suggest is a substantial improvement over current methods. Testing this hope will require the application of our framework to a variety of situations requiring risk assessment. Understanding the assumptions used in a risk assessment provides a way to interpret the risk estimates. Assumptions can be divided into those that come from the model structure and those that come from the parameter estimates. Model descriptions must provide explicit information on the assumptions used when constructing the model. For example, the standard methods for analyzing biosolids-monitoring data can generally identify some viruses (such as rotavirus, enterovirus, and reovirus) and not other viruses (such as norovirus, hepatitis, and adenoviruses). This, coupled with a recovery rate < 100%, suggests that these data are an underestimate of the actual pathogen levels in biosolids. Monitoring data, however, contain useful information provided that their limitations are understood and the data are used in an appropriate manner. These limitations are not unique to biosolids risk assessment, as all microbial risk assessments are pathogen specific and rely on occurrence data. Furthermore, if we use these data in a risk analysis, comparing the risk of one scenario with another, the fact that the resultant risk estimate is an underestimate of the true cumulative risk is less of a concern. Another assumption is associated with the fact that there is a limited amount of peer-reviewed dose–response data. However, when the available dose–response data are used in an appropriate manner, there is the potential for substantial inference. For example, one conservative approach that we employed in this work is the use of the rotavirus dose–response function for all monitored viruses, because rotavirus is the most infectious virus for which dose response data are available.

In the exposure component of our risk model, we assumed that digesters were well-mixed, first-order processes. Additionally, we made explicit assumptions about the ground-water and aerosol transport, as well as how the biosolids were applied. Parameter estimates, such as the ingestion rate and treatment efficacy, relied on data from the literature, each with its own set of assumptions. In general, the assumptions made in these risk assessments were meant to balance realism with simplicity for the purpose of demonstrating the utility of this biosolids risk framework. A site-specific risk assessment would need to collect as much site-specific information as possible to conduct a realistic risk assessment. To relax many of these model assumptions listed in Table A-2 [Supplemental Material (online at http://www.ehponline.org/members/2008/10994/suppl.pdf)] will require target data collection through either monitoring or experimental design. We hope that our work helps to motivate the collection of such necessary, targeted data in the future.

The analysis presented here provides an approach to conducting risk assessments that takes advantage of pathogen data from raw sludge and data on treatment process efficacy as additional data sources to the posttreatment data. In this context, the raw sludge and treatment data are considered prior information that can inform the risk estimate, and the post-treatment data are used to inform the likelihood. A Bayesian structure like this one can be used to take full advantage of the available data to obtain the best risk estimate. As with any Bayesian analysis, if the data informing the likelihood are sparse, the posterior (or risk estimate) is strongly influenced by the prior. As the variance of the data decreases, the posterior (or risk estimate) is influenced less by the prior.

In this study, we provided risk estimates to illustrate how this risk-modeling approach can be used both in a regulatory context to make risk-based rules and in an operational context to examine the benefits of changing treatment processes in the context of current application practices or proposed new practices. Specifically, this microbial-risk framework presented here provides a tool for *a*) biosolids producers to interpret biosolids monitoring data in the context of risk, *b*) treatment plant engineers to evaluate the potential risk-based benefits of making operational changes to existing treatment and/or adding additional treatment processes, *c*) environmental managers to evaluate potential capital improvements and/or land application site placement issues from a health-based perspective, and *d*) regulators at the U.S. EPA to develop risk-based regulations parallel to the chemical contaminant rules.

## Figures and Tables

**Table 1:** Raw sludge and treated biosolids monitoring data.

Sample month	Raw sludge enteric virus (PFU/4 g)	Treated biosolids enteric virus (PFU/4 g)
1	< 1	< 1
2	< 1	< 1
3	< 1	< 1
4	< 1	< 1
5	< 1	< 1
6	5	< 1
7	2	< 1
8	1	< 1
9	1	< 1
10	7	< 1
11	6	1
12	31	< 1

**Table 2:** Treatment data summary.

Type of anaerobic digestion	Log_10_ reduction	Reference
Mesophilic	0.5–2	Sorber and Moore 1986
Mesophilic	1	Gerba et al. 2002
Mesophilic	1	U.S. EPA 1991
Mesophilic	1.36	U.S. EPA 1991
Mesophilic	1.05	U.S. EPA 1991
Mesophilic (3 plants)	0.03, 0.01, 0.003 (all per day)	Goddard et al. 1981
Thermophilic log reduction	3	U.S. EPA 1991
Equation for inactivation based on temperature	0.420T–13.623	Hurst 1989

T, incubation temperature.

**Table 3:** Comparison of risk for one- and two-digester treatments.

	Model 1 (1 digester)	Model 2 (2 digesters)
Mean	1 × 10^−3^	2 × 10^−4^
Median	6 × 10^−5^	8 × 10^−7^
75th percentile	6 × 10^−4^	3 × 10^−5^
Pr(Risk >10^−4^)	0.46	0.16

Pr, probability of risk.
